# HMGB1: The Central Cytokine for All Lymphoid Cells

**DOI:** 10.3389/fimmu.2013.00068

**Published:** 2013-03-20

**Authors:** Guanqiao Li, Xiaoyan Liang, Michael T. Lotze

**Affiliations:** ^1^The University of Pittsburgh Cancer Institute, University of Pittsburgh School of MedicinePittsburgh, PA, USA; ^2^Department of Surgery, University of Pittsburgh School of MedicinePittsburgh, PA, USA; ^3^Department of Immunology, University of Pittsburgh School of MedicinePittsburgh, PA, USA; ^4^Department of Bioengineering, University of Pittsburgh School of MedicinePittsburgh, PA, USA

**Keywords:** lymphocytes, HMGB1, TLR2, TLR4, RAGE, NK cells, T cells, B cells

## Abstract

High-mobility group box 1 (HMGB1) is a leaderless cytokine, like the IL-1 and FGF family members, that has primary roles within the nucleus and the cytosol. Within the nucleus, it serves as another guardian of the genome, protecting it from oxidant injury and promoting access to transcriptional complexes such as nuclear hormone/nuclear hormone receptors and p53/p73 complexes. Within the cytosol it promotes autophagy and recruitment of the myddosome to Toll-like receptor (TLR) 9 vesicular compartments. Outside of the cell, it can either bind to specific receptors itself, or with high affinity to DNA, nucleosomes, IL-1β, lipopolysaccharide, and lipoteichoic acid to mediate responses in specific physiological or pathological conditions. Currently identified receptors include TLR2, TLR4, the receptor for advanced glycation end products, CD24-Siglec G/10, chemokine CXC receptor 4, and TIM-3. In terms of its effects or functions within lymphoid cells, HMGB1 is principally secreted from mature dendritic cells (DCs) to promote T-cell and B-cell reactivity and expansion and from activated natural killer cells to promote DC maturation during the afferent immune response. Some studies suggest that its primary role in the setting of chronic inflammation is to promote immunosuppression. As such, HMGB1 is a central cytokine for all lymphoid cells playing a role complementary to its better studied role in myeloid cells.

## Introduction

Damage-associated molecular pattern (DAMPs) molecules, are endogenous danger signals that elicit inflammation and subsequent immune responses once released from dead or stressed cells following injury or infection (Rubartelli and Lotze, 2007; Sims et al., 2010; Tang et al., 2012). Although various DAMPs have been identified, the best characterized is the prototypic nuclear protein high-mobility group box 1 (HMGB1). HMGB1 is an evolutionarily ancient protein that was first discovered as a chromatin-associated protein more than three decades ago (Goodwin et al., 1977). We now know that it displays many other functions depending on its location and its synergizing partners. Present within the nuclei of almost all eukaryotic cells, HMGB1 functions as a DNA chaperone that stabilizes nucleosome formation and promotes access to transcriptional factors that target specific genes (Müller et al., 2001; Thomas, 2001), although HMGB1 itself is not sequence specific. Our group demonstrated that cytosolic HMGB1 also promotes autophagy, a conserved programed survival pathway evoked following environmental and intracellular stress (Tang et al., 2010a, 2012). Apart from its nuclear and cytosolic roles, HMGB1 possesses a previously unexpected multifaceted role in immunity when released or secreted into the extracellular milieu. This occurs in two principal ways: either (1) passively released from necrotic cells (Scaffidi et al., 2002) or (2) actively secreted by inflammatory cells, such as monocytes or macrophages (Gardella et al., 2002; Bonaldi et al., 2003; Tang et al., 2007) and natural killer (NK) cells (Semino et al., 2005; Gougeon and Bras, 2011). It this way, HMGB1 evokes innate immune response via its interaction with cell surface receptors.

Previous studies highlight the importance of HMGB1 at the core of inflammation-associated events, acting as an irreplaceable modulator of immune responses and the “universal” biosensor for nucleic acids (Yanai et al., 2012). In spite of its well-established divergent functions in myeloid cells which predominantly participate in innate immune response, its roles in adaptive immunity involving T-cells and B-cells is so far not fully understood and surprisingly, one which needs substantially more study. Here, we describe the cytokine-like biology of HMGB1 protein, with a focus on lymphoid cells, including NK cells, T-cells, and B-cells.

## Lessons from HMGB1 Knockouts

High-mobility group box 1 is vital for *ex utero* growth, as shown by inborn defects and rapid death (within 24 h following birth) in *hmgb1^−/−^* mice, as early as E15 in inbred species, because of hypoglycemia. This was initially postulated to be the result from deficient glucocorticoids receptor function (Calogero et al., 1999), but we would now attribute this to reduced autophagy, critically important for survival in the neonatal period (Kuma et al., 2004). Necrotic HMGB1^−/−^ cells only weakly activate dendritic cells (DCs) (Rovere-Querini et al., 2004), and HMGB1-deficient DCs display sharply impaired capacity to trigger inflammation (Scaffidi et al., 2002). We now know that floxed HMGB1 deleted in a tissue- or cell type-specific fashion within the pancreas, liver, small bowel, DCs, and NK cells, is associated with prolonged viability of animals compared with complete knockout of HMGB1 (unpublished observations) in the whole animal, suggesting that these are not the target tissues associated with lethality.

## HMGB1 as the Cytokine for Lymphoid Cells

High-mobility group box 1 was identified as a delayed mediator of inflammation released from macrophages (Wang et al., 1999), found in the serum 24–48 h later than secretion IL-1β and tumor necrosis factor (TNF)-α, the classical early pro-inflammatory cytokines which are dissipated by 24 h. Afterwards, it was demonstrated to be liberated from cells undergoing necrosis, followed by production of TNF-α from monocytes (Scaffidi et al., 2002). Subsequent investigations uncovered an amazingly profligate role in mediating local or systemic immune responses through its interaction with several receptors. As a cytokine, it transduces signals and coordinates cellular activities through several pattern-recognition receptors including the receptor for advanced glycation end products (RAGE), Toll-like receptor (TLR)2, TLR4, TIM-3, chemokine CXC receptor (CXCR)4, CD24-Siglec G/10 (Park et al., 2004, 2006; Dumitriu et al., 2005; Lotze and Tracey, 2005; Bianchi, 2009; Chen et al., 2009; Tang and Lotze, 2012; Tang et al., 2012; Yanai et al., 2012), and TLR-9 when combined with DNA (Tian et al., 2007). Extracellular HMGB1 thus functions as a modulator, modifying the immunogenic potentials of DNA and potentially other PAMPs and DAMPs and cytokines. Indeed, given the differences in the all thiol form of HMGB1, promoting primarily chemokine activity and the dithiol form which promotes TNF/IL-6 production (cytokine activity), it is quite likely that the molecule secreted by activated cells, endowed with autocrine and paracrine actions, differs biochemically and functionally from the molecule released as a consequence of cell and tissue necrosis (Venereau et al., 2012). Given this difference with the all thiol form promoting release of the chemokine CXCL12, and the dithiol not, environmental conditions likely dictate the eventual outcome of HMGB1 interactions with lymphoid cells in the tissues. For example, well perfused and non-hypoxic environments may promote different T-cell responses that hypoxic, reducing conditions (Venereau et al., 2012). TLRs, the best-studied pattern-recognition receptors (PRRs), are highly conserved proteins initiating immune responses following recognition of various molecules derived from pathogens (PAMPs) as well as endogenous danger signals (DAMPs) sharing similar structures (Medzhitov and Janeway, 1997; Aderem and Ulevitch, 2000; Medzhitov, 2001). The intracellular signaling cascades after recognition principally involve two specific adaptors, the Toll/IL-1R domain-containing adaptor TRIF and myeloid differentiation primary response protein (MyD88), which is primarily involved in HMGB1-mediated signaling pathway and acts as a component of myddosome with IRAK2 and IRAK4 assembled in response to primary stimulation (Motshwene et al., 2009; Lin et al., 2010; George et al., 2011). RAGE is a PRR with a wide variety of ligands including advanced glycation end products (AGEs) and DAMPs (Sparvero et al., 2009; Sims et al., 2010). The list of receptors that interact with HMGB1 continues to grow, as does interest in understanding the signaling pathways and their cooperative functions in specific cell types. Current insights on these receptors, based on experimental observations, is that TLRs principally are involved in the activation of myeloid cells, whereas RAGE is primarily activated in endothelial and somatic cells (Yanai et al., 2012).

**Figure 1:**
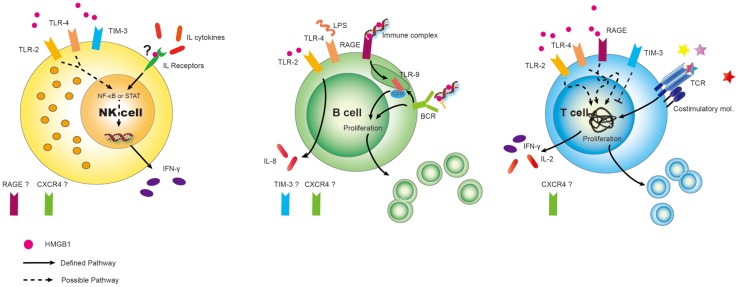
**Differential expression of DAMP receptors for HMGB1 on lymphoid cells**. Surprisingly, given the surfeit of receptors now shown to be expressed on various cell types, relatively little has been done to fully characterize the panoply of receptors on lymphoid cells. Best defined are the expression of RAGE on Ba and T-cells (but not to our knowledge on NK cells). Similarly TLR2, TLR4, CXCR4, and possible TIM-3 could play critical roles on subserving cell functions in response to HMGB1 on immune cells and this needs to be more carefully studied. Possible and defined roles are shown. “?” represents we hypothesize that HMGB1 binds to IL receptors, assisting in interleukin (cytokine) interaction. Also, expression of other HMGB1 receptors are unknown at present.

High-mobility group box 1 signaling has been studied in many cell types following interaction with individual receptors, with most studies centering on myeloid cells – the maturation of conventional DCs, their role in plasmacytoid DCs, activation of monocytes or macrophages, and the production of pro-inflammatory cytokines (Lotze and Tracey, 2005; Yang et al., 2007; Bianchi, 2009; Yanai et al., 2012). Their effects on lymphoid cells, however, are surprisingly not well characterized. We have extraordinarily limited information about the expression of receptors RAGE and, TLR2/4 and TIM-3 on both helper and regulatory T-cells (Wild et al., 2012), RAGE and TLR2/4/9 on B-cells (Tian et al., 2007; Avalos et al., 2010), and TLR2/4 and TIM-3 on NK cells (Tang and Lotze, 2012), shown in Figure 1. Beyond that there is quite little information. In this review, we summarize the critical roles of HMGB1 in lymphoid cells (Table 1), with a focus on its extracellular role acting as a cytokine.

**Table 1:** **Lymphoid cells respond to HMGB1**.

	NK cells	B-cells	T-cells
Nuclear	Modulation of transcriptional activity of various genes, including steroid hormone receptors, NF-κB, p53/p73 transcriptional complexes, and some homeobox-containing protein (Erlandsson Harris and Andersson, 2004; Lotze and Tracey, 2005)		
		Nuclear assistance in assembly of recombination activating gene 1/2(RAG1/2)-DNA complex for V(D)J recombination of B-cell receptors (BCR) and T-cell receptors (TCR) (Agrawal and Schatz, 1997; Dai et al., 2005)	

Cytosolic	Regulation of autophagy (Tang et al., 2010a) (other cell types, not discovered in lymphoid cells yet)		
		Recruitment of MyD88 to TLR-9 (Ivanov et al., 2007)	
	Universal biosensor of nucleic acid (Yanai et al., 2012)		

Extracellular	Synergy with other cytokines to modulate cell functions via binding cytokine receptors (CXCR4 for example)		
	Increased IFN-γ secretion in macrophage-stimulated NK cells (DeMarco et al., 2005)	Activation and proliferation in the form of immune complex (HMGB1 + DNA) (Tian et al., 2007; Avalos et al., 2010)	Expansion, activation, and polarization of Th1 cells (Messmer et al., 2004; Dumitriu and Baruah, 2005; Sundberg et al., 2009)
		Spontaneous IL-8 production (McDonnell et al., 2011)	Infiltration of T-cells expressing lymphotoxin and tumor progression (He et al., 2012a)

**Figure 2:**
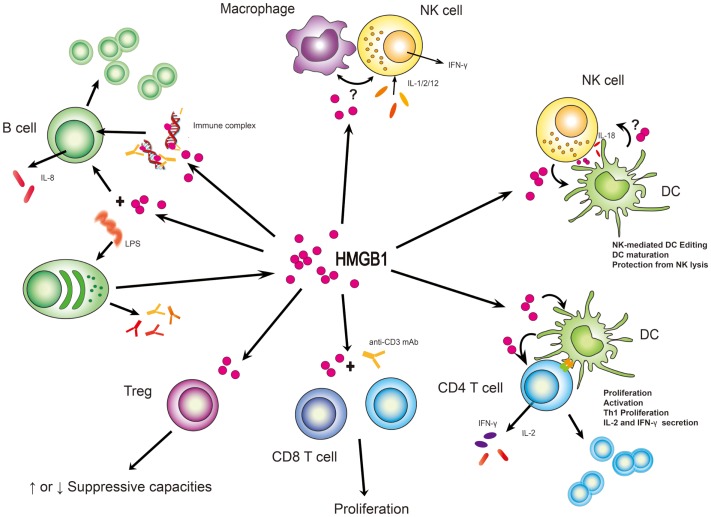
**HMGB1 stimulates effector function from immune cells**. In many instances, HMGB1’s function has been to subserve cell:cell interactions between lymphoid and myeloid cells. Its clearest definition has been with NK/macrophage or NK/DC interactions although suspected roles in B and T-cell activities are supported. What is unclear is whether these roles are cell type specific and whether HMGB1 might act in trans between a myeloid and lymphoid cell. “?” represents the state of uncertainty if HMGB1 plays a role in the setting of macrophage and NK cell interaction or HMGB1 is secreted from DCs to affect NK cell function.

Most cytokines function distinctly in synergy or antagonism with other cytokines acting collectively. This is also true for HMGB1. Moreover, HMGB1 shares pleiotropic and redundant characteristics with other cytokines (Lotze and Tracey, 2005), sometimes binding them to enhance immunologic function, thereby endowing them with a more potent capacity to elicit biological and immunological responses, consequences depending on the local microenvironmental factors and presence of other circumstances. Here, we list out different cellular responses of lymphoid cells to HMGB1 in different conditions or settings (Table 2) which could act as a reference for readers to make comparisons or conduct experiments. Also, we summarize the common consequences in response to HMGB1 (Figure 2).

**Table 2:** **Cellular responses of lymphoid cells to HMGB1 located differently**.

Cell types	Species	Disease	HMGB1 effect	Stimulation	Secreted from	Receptors	Summary	Reference
T-cell expressing lymphotoxin	Mouse	Prostate cancer		Specific antigens	Cancer cells or inflammatory cells (?)		HMGB1 is required for infiltration and activation of antigen-experienced T-cells expressing lymphotoxin α1β2(LT), but not helper or regulatory T-cells, followed by recruitment of macrophages to the tumor site in an LTβR-dependent manner, thus prompting tumor malignant progression	He et al. (2012a)
CD4 T-cell	Human	–	Direct	Activated or α-CD3/α-CD28 Abs	Endotoxin-stimulated DC	RAGE on DC	HMGB1 is translocated and secreted by human DC upon stimulation, maintaining itself maturation, and improving CD4+ T-cell expansion, survival, and Th1 polarization. Blockade with anti-HMGB1 Abs or Box A, the effect is drastically impaired. However, T-cell activation cannot be stimulated by HMGB1 alone, but also requires Ag receptor and co-stimulatory signals (CD3 and CD8 crosslinking mimics the event *in vitro*)	Dumitriu and Baruah (2005)
CD4 T-cell	Human	–	Indirect	HMGB1-stimulated DC		RAGE on DC	HMGB1 as well as B box trigger phenotypic maturation and pro-inflammatory cytokine secretion via both RAGE-mediated NF-κB and p38 MAPK pathway. And activated DC will further drive Th1 polarization, as evidenced by secretion of IL-2 and IFN-γ	Messmer et al. (2004)
CD4 CD8 T-cell	Human	–	Direct	α-CD3 mAb			HMGB1 behaves as a proliferative signal for both human CD4 and CD8 T-cells in response to suboptimal anti-CD3 mAb stimulation	Sundberg et al. (2009)
CD4 Treg Tcon	Human	–	Direct	TCR/co-stimulation (CD2/CD3/CD28 beads)		RAGE TLR4	HMGB1 prompts survival and suppressive capacities of Treg in a RAGE-mediated fashion, whereas suppresses IFNγ release of Tcon (conventional) and inhibits their proliferation via TLR4, indicating that TCR/co-stimulatory signal is abrogated by HMGB1	Wild et al. (2012)
CD4 T-cell↓	Mouse		Indirect	CD11C^low^ CD45RB^high^ DC			IL-10 producing CD11C^low^ CD45RB^high^ mouse DCs display mature phenotype and secrete IL-10 upon HMGB1 stimulation in a dose-dependent manner, therefore potentially diminish T-cell response and driving Th2 polarization	Liu et al. (2011)
CD4 T-cell	Rat	Burn	Direct				HMGB1 markedly limits the proliferation of rat T-cells during post-burn, consistent with decreased expression of IL-2 and IL-2Rα. T-cells polarized to Th2 after HMGB1 stimulation *in vivo*	Zhang et al. (2008)
CTL (CD8 T-cell)	Human mouse		Indirect	DC + dying tumor cells	Dying tumor cells	TLR4/MyD88 on DC	In the context of chemo- or radio-therapy, functional binding between HMGB1 released by dying cells and its receptor TLR4 on DC is prerequisite for efficient antigen presentation of tumor antigens and induction of CTL immunity	Apetoh et al. (2007)
Treg	Mouse		Direct			TLR4	HMGB1 modulates the suppressive capacity of Treg through TLR4-dependent pathway. The expression level of CTLA4 and Foxp3 in Treg cells as well as IL-10 secretion were significantly diminished after HMGB1-treatment, which was restored by administration of anti-TLR4 antibody	Zhu et al. (2011)
Autoreactive B-cell	Mouse	SLE	Direct	Immune complex (+CpG)		TLR-9 and RAGE	HMGB1 acts to activate pDCs and IgG2a-reactive B-cell receptor (BCR) transgenic B-cells in form of DNA-containing immune complex via TLR-9-dependent pathway. The response is considerately elicited with the help of surface RAGE	Tian et al. (2007)
Autoreactive B-cell	Mouse	SLE	Direct	Immune complex (+DNA)		TLR-9 and BCR Not RAGE	B-cells can undergo activation and proliferation in response to chromatin immune complexes (ICs) containing HMGB1-DNA in a TLR-9-mediated manner by specific antibody engagement of BCR but not RAGE	Avalos et al. (2010)
B-cell	Human	Inflammatory bowel disease	Direct	±LPS		TLR2 and CD36	Endogenous HMGB1 induces B-cell activation through TLR2 and CD36, whereas exogenous endotoxin may exhibit disease-specific effects on B-cells, unexpectedly evoking pro- or anti-inflammatory responses. Moreover, serum levels of HMGB1 are linked with spontaneous IL-8 production	McDonnell et al. (2011)
B-cells	Mouse			LPS	LPS-stimulated splenic plasma cell		Non-canonical inflammatory cytokine HMG-1 is released from plasma cells into the extracellular milieu following B-cell maturation, demonstrating its pro-inflammatory role	Vettermann et al. (2011)
NK cells	Human		Direct/indirect	+IL-2/1/12 + monocyte			HMGB1 in concert with IL-2 and IL-1 or IL-12 facilitates interferon gamma release from macrophage-stimulated NK cells	DeMarco et al. (2005)

## HMGB1 and NK Cells

There is little information about the direct effects of HMGB1 on NK cells, with the exception of elevated secretion of IFN-γ by macrophage-stimulated NK cells in concert with other pro-inflammatory cytokines like IL-2 and IL-12 (DeMarco et al., 2005), work done by our group almost a decade ago. Nevertheless, whether it occurs depending on the activation of monocytes or in a direct NK-cell specific manner is still unclear. Further clarification as to which cell type is responsive to HMGB1 under these specific circumstance is needed and of significant interest, since little is known about the interaction between monocytes (macrophages) and NK cells, both of which are key sentinels and instigators of immune responses.

Natural killer cells do, however, secrete HMGB1. HMGB1 undergoes abundant, regulated secretion from activated NK cells into the immunological synapse during NK/iDC (immature DC) crosstalk, thus inducing maturation of DCs and limiting NK cell-mediated cytotoxicity of the DCs (Semino et al., 2005). The secretion of HMGB1 is markedly elevated following engagement of NKp30 (one type of natural cytotoxicity receptor or NCR) expressed on human NK cells, thereby triggering maturation of autologous DC (Semino et al., 2007). Whether or not DC can in turn secrete HMGB1 for further activation of NK cells and promote the quality of the crosstalk remains undefined. Specific cell types without HMGB1 expression would be needed to uncover the critical role of HMGB1 in intracellular communication.

High-mobility group box 1-mediated NK/DC crosstalk is important in the setting of HIV infection (Saïdi et al., 2008; Melki et al., 2010; Gougeon and Bras, 2011). DCHIV (infected by HIV virus) are resistant to the NK cell-induced editing process. Interestingly, HMGB1, essential for DC maturation presumably within secondary lymphoid tissues, also contributes to viral replication and DC persistence via up-regulation of apoptosis inhibitors against TRAIL (TNF-related apoptosis-inducing ligand)-mediated apoptosis.

Given that the cooperative dialog between NK cells and DCs is pivotal for sustaining innate immunity and initiating the subsequent adaptive immune response, it is worth investigating the detailed mechanism by which NK/DC crosstalk and its altered processes link to clinical manifestations of diseases, including cancer, autoimmune, and infectious diseases.

## HMGB1 and T-Cells

Our knowledge of HMGB1 effector functions to T-cells are principally based on observations and inferences from the evaluation of T-cell subsets with the treatment of HMGB1 co-cultured with DCs. In the presence of other cytokines, HMGB1 can modify the fate of the overall immune response, promoting immunity or tolerance as demonstrated by targeting effector T-cells and regulatory T-cells (Tregs) reciprocally in response to individual stimuli. Differences that may have been found in terms of immunity or tolerance when comparing various observations might be, at least in part, due to differences in the experimental systems utilized (dosage, duration, and the presence of other factors) and the pharmacological inhibitors utilized to block these complex biological systems *in vitro* and *in vivo*. We know little about the effects of HMGB1 on naïve and memory T-cells as regards alteration in phenotype or cytokine proficiency among the defined T-cell subsets. In addition, the ability of HMGB1 to either recruit T-cells to sites of tissue damage or injury, thus allowing effector T-cell function, or to induce Treg infiltration and expansion is largely unknown.

### Direct effects on T-cells

Acting as a pro-inflammatory cytokine, HMGB1, is not only released by stressed or necrotic tissues but also translocated and secreted by human DC following PAMP [endotoxin/lipopolysaccharide (LPS)] stimulation. It plays a critical role in promoting expansion, survival, and helper T (Th) 1 polarization of CD4+ T-cells (Dumitriu and Baruah, 2005; Jube et al., 2012). Similarly, HMGB1, is also a proliferative signal for both human CD4+ and CD8+ T-cells in response to suboptimal anti-CD3 mAb stimulation (Sundberg et al., 2009). The expression level of CTLA4 and Foxp3 in Treg cells as well as IL-10 secretion are significantly diminished following HMGB1 treatment. This is restored by administration of an anti-TLR4 antibody (Zhu et al., 2011). Altogether, HMGB1 is seemingly necessary for enhancing immunity through activation of effector T-cells and suppression of Treg’s. In contrast, HMGB1 can also promote migration and survival of Treg, whereas it suppresses IFNγ release of conventional T-cells and inhibits their proliferation via TLR4, indicating that the TCR/co-stimulatory signal is abrogated by HMGB1. Furthermore, HMGB1 elicits increased suppressive capacity of Treg when co-cultured with effector T-cells in a RAGE-dependent fashion. Additionally, several reports provide evidence suggesting that HMGB1 may contribute to Th17 cells proliferation and activation in the context of autoimmune disease, including rheumatoid arthritis, myocarditis, as well as acute allograft rejection (Duan et al., 2011; Su et al., 2011; He et al., 2012b; Shi et al., 2012).

When we examine immune responses *in vivo*, the findings are totally different. HMGB1 is essential for infiltration and activation of T-cells expressing lymphotoxin α1β2(LT) in mice with prostate cancer, therefore recruiting macrophages to promote tumor malignant progression (He et al., 2012a). This work further confirms the notion that HMGB1 can prompt progression of many types of cancers (Tang et al., 2010b). Surprisingly, neither T effectors nor Tregs are detected differentially between normal and cancerous tissues. The source of extracellular HMGB1 needs to be further characterized, whether arising from stressed tumor cells or recruited inflammatory cells, including NK cells or DCs, or all. HMGB1 markedly limits the proliferation of murine (rat) T-cells and induces Th2 polarization following burn injury, consistent with decreased expression of IL-2 and IL-2Rα (Zhang et al., 2008).

### DC-mediated indirect effects on T-cells

High-mobility group box 1 is an inducer of DC maturation (Messmer et al., 2004; Rovere-Querini et al., 2004; Semino et al., 2005, 2007). Mature and activated DC will further drive Th1 polarization, as evidenced by secretion of IL-2 and IFN-γ (Messmer et al., 2004). It is worth noting that one of the promising mechanisms underlying the chemo- or radio-therapy-based antitumor responses is due to the functional binding between HMGB1 released by dying cells and one of its receptors, TLR4 expressed on DC, which allows for antigen presentation and subsequent cytotoxic CD8+ T-cell (CTL) effector function (Apetoh et al., 2007).

On the other hand, IL-10 producing CD11C^low^ CD45RB^high^ mouse DCs also display a mature phenotype and secrete IL-10 following HMGB1 stimulation in a dose-dependent manner, thereby potentially diminishing T-cell responses with down-regulation of IL-2 and IL-2Rα and driving Th2 polarization, just the opposite in the case of CD11C^high^ CD45RB^low^ DCs. This finding is in concordance with the potential of HMGB1 to polarize Th2 cells in rats following thermal injury (Zhang et al., 2008).

## HMGB1 and B-Cells

Compared with T-cells, the role of HMGB1 in B-cells has not been fully delineated. Some studies have supported a role for HMGB1 in B-cell activation. In the form of immune complexes (ICs), HMGB1 promotes proliferation of autoreactive B-cells in response to endogenous TLR-9 ligands (e.g., DNA) (Tian et al., 2007; Avalos et al., 2010). This suggests are markedly immune-regulatory function in the pathogenesis of autoimmune diseases. TLR-9 is responsive to immune complex in intracellular endosomes, while the internalization of DNA may be mediated by RAGE which bound with HMGB1 (Tian et al., 2007) or by specific IgG and B-cell receptor interaction, followed by BCR engagement (Avalos et al., 2010). However, given that B-cell proliferation and Ig gene recombination share the same pathway but contrary states of molecules involved (e.g., FOXO degradation or dephosphorylation) and autoreactive antigen, the capacity of antibody production could be further investigated in terms of individual receptors of IC interaction, thus providing a comprehensive role for HMGB1 in B-cell activation. Furthermore, in the context of inflammatory bowel disease (IBD), enhanced serum levels of HMGB1 is accompanied by spontaneous IL-8 production by B-cells via interaction with TLR2 and CD36 (McDonnell et al., 2011). On the other hand, plasma cells release HMGB1 into the extracellular milieu following LPS-stimulated maturation (Vettermann et al., 2011), demonstrating its pro-inflammatory effects in promoting autoimmune disease and chronic inflammation.

## Concluding Remarks

High-mobility group box 1, like other cytokines, is able to function as an agonist, an antagonist, to synergize with other factors and to have multiple pleiotropic functions on multiple cell types, including lymphoid cells. Unlike typical cytokines however, it interacts with a panoply of receptors, many of which are notably promiscuous with functions quite disparate from each other, depending upon the local microenvironment, location, and coordination with individual stimuli. In addition, unlike cytokines which interact with picogram or nanogram quantities to promote full receptor activation, HMGB1 requires, in many instances, microgram quantities in order to elicit a meaningful response *in vitro*. Increasing advances in understanding the role of HMGB1 in immunity have extended the knowledge and led to widespread acceptance of the notion that HMGB1 acts as a centrally important, potent, ubiquitous cytokine which exerts effect on both myeloid and lymphoid cells. It thus plays a multifaceted modulatory role in both innate and adaptive immune responses. Although there is much information about the diverse, sometimes even opposite effects of HMGB1 on various kinds of immune cells in culture, it is of great importance to understand the precise mechanism by which HMGB1 functions *in vivo*, in particular during altered pathology or physiology. In a complicated balance of guiding and choreographing disparate biologies, HMGB1, interspersed with DAMPs and PAMPs, develops the plot line and provides impetus to the emergent immune response. Improved understanding of when, where, which cell types produce/respond to HMGB1 and what levels at intimate cell:cell contact or released into tissues or systemically would provide a basis for suitable therapeutic implementation or interventions in the clinic.

## Conflict of Interest Statement

The authors declare that the research was conducted in the absence of any commercial or financial relationships that could be construed as a potential conflict of interest.

## References

[B1] Aderem A. Ulevitch R. (2000). Toll-like receptors in the induction of the innate immune response. Nature 406 Available at: http://gene.bjmu.edu.cn/undereducation_education/download/Toll-like receptor in the induction of the innate immune response.pdf [accessed December 5, 2012]. 10.1038/35021228 10963608

[B2] Agrawal A. Schatz D. G. (1997). RAG1 and RAG2 form a stable postcleavage synaptic complex with DNA containing signal ends in V(D)J recombination. Cell 89, 43–53 10.1016/S0092-8674(00)80181-6 9094713

[B3] Apetoh L. Ghiringhelli F. Tesniere A. Obeid M. Ortiz C. Criollo A. (2007). Toll-like receptor 4-dependent contribution of the immune system to anticancer chemotherapy and radiotherapy. Nat. Med. 13, 1050–1059 10.1038/nm1622 17704786

[B4] Avalos A. M. Kiefer K. Tian J. Christensen S. Shlomchik M. Coyle A. J. (2010). RAGE-independent autoreactive B cell activation in response to chromatin and HMGB1/DNA immune complexes. Autoimmunity 43, 103–110 10.3109/08916930903384591 20014975 PMC2929824

[B5] Bianchi M. E. (2009). HMGB1 loves company. J. Leukoc. Biol. 86, 573–576 10.1189/jlb.0309121 19414536

[B6] Bonaldi T. Talamo F. Scaffidi P. Ferrera D. Porto A. Bachi A. (2003). Monocytic cells hyperacetylate chromatin protein HMGB1 to redirect it towards secretion. EMBO J. 22, 5551–5560 10.1093/emboj/cdg516 14532127 PMC213771

[B7] Calogero S. Grassi F. Aguzzi A. Voigtländer T. Ferrier P. Ferrari S. (1999). The lack of chromosomal protein Hmg1 does not disrupt cell growth but causes lethal hypoglycaemia in newborn mice. Nat. Genet. 22, 276–280 10.1038/10338 10391216

[B8] Chen G.-Y. Tang J. Zheng P. Liu Y. (2009). CD24 and Siglec-10 selectively repress tissue damage-induced immune responses. Science 323, 1722–1725 10.1126/science.1169399 19264983 PMC2765686

[B9] Dai Y. Wong B. Yen Y. Oettinger M. A. Kwon J. Johnson R. C. (2005). Determinants of HMGB proteins required to promote RAG1/2-recombination signal sequence complex assembly and catalysis during V(D)J recombination. Mol. Cell. Biol. 25, 4413–4425 10.1128/MCB.25.22.9936-9948.2005 15899848 PMC1140611

[B10] DeMarco R. A. Fink M. P. Lotze M. T. (2005). Monocytes promote natural killer cell interferon gamma production in response to the endogenous danger signal HMGB1. Mol. Immunol. 42, 433–444 10.1016/j.molimm.2004.07.023 15607795

[B11] Duan L. Wang C.-Y. Chen J. Gong Q. Zhu P. Zheng F. (2011). High-mobility group box 1 promotes early acute allograft rejection by enhancing IL-6-dependent Th17 alloreactive response. Lab. Invest. 91, 43–53 10.1038/labinvest.2010.141 20714327

[B12] Dumitriu I. Baruah P. (2005). Release of high mobility group box 1 by dendritic cells controls T cell activation via the receptor for advanced glycation end products. J. Immunol. 174, 7506–7515 15944249 10.4049/jimmunol.174.12.7506

[B13] Dumitriu I. E. Baruah P. Bianchi M. E. Manfredi A. A. Rovere-Querini P. (2005). Requirement of HMGB1 and RAGE for the maturation of human plasmacytoid dendritic cells. Eur. J. Immunol. 35, 2184–2190 10.1002/eji.200526066 15915542

[B14] Erlandsson Harris H. Andersson U. (2004). Mini-review: the nuclear protein HMGB1 as a proinflammatory mediator. Eur. J. Immunol. 34, 1503–1512 10.1002/eji.200424916 15162419

[B15] Gardella S. Andrei C. Ferrera D. Lotti L. (2002). The nuclear protein HMGB1 is secreted by monocytes via a non-classical, vesicle-mediated secretory pathway. EMBO Rep. 3, 995–1001 10.1093/embo-reports/kvf198 12231511 PMC1307617

[B16] George J. Motshwene P. G. Wang H. Kubarenko A. V. Rautanen A. Mills T. C. (2011). Two human MYD88 variants, S34Y and R98C, interfere with MyD88-IRAK4-myddosome assembly. J. Biol. Chem. 286, 1341–1353 10.1074/jbc.M110.159996 20966070 PMC3020742

[B17] Goodwin G. Rabbani A. Nicolas P. Johns E. (1977). The isolation of the high mobility group non-histone chromosomal protein HMG 14. FEBS Lett. 80, 413–416 10.1016/0014-5793(77)80488-2 891994

[B18] Gougeon M.-L. Bras M. (2011). Natural killer cells, dendritic cells, and the alarmin high-mobility group box 1 protein: a dangerous trio in HIV-1 infection? Curr. Opin. HIV AIDS 6, 364–372 10.1097/COH.0b013e328349b089 21825870

[B19] He Y. Zha J. Wang Y. Liu W. Yang X. Yu P. (2012a). Tissue damage-associated “danger signals” influence T cell responses that promote the progression of pre-neoplasia to cancer. Cancer Res. Available at: http://www.ncbi.nlm.nih.gov/pubmed/23108142 [accessed November 17, 2012]. 10.1158/0008-5472.CAN-12-2704 23108142

[B20] He Z. Shotorbani S. S. Jiao Z. Su Z. Tong J. Liu Y. (2012b). HMGB1 promotes the differentiation of Th17 via up-regulating TLR2 and IL-23 of CD14+ monocytes from patients with rheumatoid arthritis. Scand. J. Immunol. 76, 483–490 10.1111/j.1365-3083.2012.02759.x 22809173

[B21] Ivanov S. Dragoi A.-M. Wang X. Dallacosta C. Louten J. Musco G. (2007). A novel role for HMGB1 in TLR9-mediated inflammatory responses to CpG-DNA. Blood 110, 1970–1981 10.1182/blood-2006-09-044776 17548579 PMC1976374

[B22] Jube S. Rivera Z. S. Bianchi M. E. Powers A. Wang E. Pagano I. (2012). Cancer cell secretion of the DAMP protein HMGB1 supports progression in malignant mesothelioma. Cancer Res. 72, 3290–3301 10.1158/1538-7445.AM2012-3290 22552293 PMC3389268

[B23] Kuma A. Hatano M. Matsui M. Yamamoto A. Nakaya H. Yoshimori T. (2004). The role of autophagy during the early neonatal starvation period. Nature 432, 1032–1036 10.1038/nature03029 15525940

[B24] Lin S.-C. Lo Y.-C. Wu H. (2010). Helical assembly in the MyD88-IRAK4-IRAK2 complex in TLR/IL-1R signalling. Nature 465, 885–890 10.1038/nature09121 20485341 PMC2888693

[B25] Liu Q. Yao Y. Yan Y. Dong N. Sheng Z. (2011). High mobility group box 1 protein suppresses T cell-mediated immunity via CD11c(low)CD45RB(high) dendritic cell differentiation. Cytokine 54, 205–211 10.1016/j.cyto.2011.01.008 21296590

[B26] Lotze M. T. Tracey K. J. (2005). High-mobility group box 1 protein (HMGB1): nuclear weapon in the immune arsenal. Nat. Rev. Immunol. 5, 331–342 10.1038/nri1594 15803152

[B27] McDonnell M. Liang Y. Noronha A. Coukos J. Kasper D. L. Farraye F. A. (2011). Systemic Toll-like receptor ligands modify B-cell responses in human inflammatory bowel disease. Inflamm. Bowel Dis. 17, 298–307 10.1002/ibd.21424 20806343

[B28] Medzhitov R. (2001). Toll-like receptors and innate immunity. Nat. Rev. Immunol. 1, 135–145 10.1038/35100529 11905821

[B29] Medzhitov R. Janeway C. A. (1997). Innate immunity: the virtues of a nonclonal system of recognition. Cell 91, 295–298 10.1016/S0092-8674(00)80412-2 9363937

[B30] Melki M.-T. Saïdi H. Dufour A. Olivo-Marin J.-C. Gougeon M.-L. (2010). Escape of HIV-1-infected dendritic cells from TRAIL-mediated NK cell cytotoxicity during NK-DC cross-talk – a pivotal role of HMGB1. PLoS Pathog. 6:e1000862 10.1371/journal.ppat.1000862 20419158 PMC2855334

[B31] Messmer D. Yang H. Telusma G. Knoll F. Li J. Messmer B. (2004). High mobility group box protein 1: an endogenous signal for dendritic cell maturation and Th1 polarization. J. Immunol. 173, 307–313 15210788 10.4049/jimmunol.173.1.307

[B32] Motshwene P. G. Moncrieffe M. C. Grossmann J. G. Kao C. Ayaluru M. Sandercock A. M. (2009). An oligomeric signaling platform formed by the Toll-like receptor signal transducers MyD88 and IRAK-4. J. Biol. Chem. 284, 25404–25411 10.1074/jbc.M109.022392 19592493 PMC2757241

[B33] Müller S. Scaffidi P. Degryse B. Bonaldi T. Ronfani L. Agresti A. (2001). New EMBO members’ review: the double life of HMGB1 chromatin protein: architectural factor and extracellular signal. EMBO J. 20, 4337–4340 10.1093/emboj/20.16.4337 11500360 PMC125571

[B34] Park J. S. Gamboni-Robertson F. He Q. Svetkauskaite D. Kim J.-Y. Strassheim D. (2006). High mobility group box 1 protein interacts with multiple Toll-like receptors. Am. J. Physiol. Cell Physiol. 290, C917–C924 10.1152/ajpcell.00401.2005 16267105

[B35] Park J. S. Svetkauskaite D. He Q. Kim J.-Y. Strassheim D. Ishizaka A. (2004). Involvement of toll-like receptors 2 and 4 in cellular activation by high mobility group box 1 protein. J. Biol. Chem. 279, 7370–7377 10.1074/jbc.M311418200 14660645

[B36] Rovere-Querini P. Capobianco A. Scaffidi P. Valentinis B. Catalanotti F. Giazzon M. (2004). HMGB1 is an endogenous immune adjuvant released by necrotic cells. EMBO Rep. 5, 825–830 10.1038/sj.embor.7400205 15272298 PMC1299116

[B37] Rubartelli A. Lotze M. T. (2007). Inside, outside, upside down: damage-associated molecular-pattern molecules (DAMPs) and redox. Trends Immunol. 28, 429–436 10.1016/j.it.2007.08.004 17845865

[B38] Saïdi H. Melki M.-T. Gougeon M.-L. (2008). HMGB1-dependent triggering of HIV-1 replication and persistence in dendritic cells as a consequence of NK-DC cross-talk. PLoS ONE 3:e3601 10.1371/journal.pone.0003601 18974890 PMC2571988

[B39] Scaffidi P. Misteli T. Bianchi M. E. (2002). Release of chromatin protein HMGB1 by necrotic cells triggers inflammation. Nature 418, 191–195 10.1038/nature00858 12110890

[B40] Semino C. Angelini G. Poggi A. Rubartelli A. (2005). NK/iDC interaction results in IL-18 secretion by DCs at the synaptic cleft followed by NK cell activation and release of the DC maturation factor HMGB1. Blood 106, 609–616 10.1182/blood-2004-10-3906 15802534

[B41] Semino C. Ceccarelli J. Lotti L. V. Torrisi M. R. Angelini G. Rubartelli A. (2007). The maturation potential of NK cell clones toward autologous dendritic cells correlates with HMGB1 secretion. J. Leukoc. Biol. 81, 92–99 10.1189/jlb.0306172 16997859

[B42] Shi Y. Sandoghchian Shotorbani S. Su Z. Liu Y. Tong J. Zheng D. (2012). Enhanced HMGB1 expression may contribute to Th17 cells activation in rheumatoid arthritis. Clin. Dev. Immunol. 2012, 295081 10.1155/2012/295081 22110531 PMC3205666

[B43] Sims G. P. Rowe D. C. Rietdijk S. T. Herbst R. Coyle A. J. (2010). HMGB1 and RAGE in inflammation and cancer. Annu. Rev. Immunol. 28, 367–388 10.1146/annurev.immunol.021908.132603 20192808

[B44] Sparvero L. J. Asafu-Adjei D. Kang R. Tang D. Amin N. Im J. (2009). RAGE (Receptor for Advanced Glycation End products), RAGE ligands, and their role in cancer and inflammation. J. Transl. Med. 7, 17 10.1186/1479-5876-7-17 19292913 PMC2666642

[B45] Su Z. Sun C. Zhou C. Liu Y. Zhu H. Sandoghchian S. (2011). HMGB1 blockade attenuates experimental autoimmune myocarditis and suppresses Th17-cell expansion. Eur. J. Immunol. 41, 3586–3595 10.1002/eji.201141879 21928275

[B46] Sundberg E. Fasth A. E. R. Palmblad K. Harris H. E. Andersson U. (2009). High mobility group box chromosomal protein 1 acts as a proliferation signal for activated T lymphocytes. Immunobiology 214, 303–309 10.1016/j.imbio.2008.09.006 19201506

[B47] Tang D. Kang R. Coyne C. B. Zeh H. J. Lotze M. T. (2012). PAMPs and DAMPs: signal 0s that spur autophagy and immunity. Immunol. Rev. 249, 158–175 10.1111/j.1600-065X.2012.01146.x 22889221 PMC3662247

[B48] Tang D. Kang R. Livesey K. M. Cheh C.-W. Farkas A. Loughran P. (2010a). Endogenous HMGB1 regulates autophagy. J. Cell Biol. 190, 881–892 10.1083/jcb.200911078 20819940 PMC2935581

[B49] Tang D. Kang R. Zeh H. J. Lotze M. T. (2010b). High-mobility group box 1 and cancer. Biochim. Biophys. Acta 1799, 131–140 10.1016/j.bbagrm.2009.11.014 20123075 PMC2818552

[B50] Tang D. Lotze M. T. (2012). Tumor immunity times out: TIM-3 and HMGB1. Nat. Immunol. 13, 808–810 10.1038/ni.2396 22910384 PMC3672065

[B51] Tang D. Shi Y. Kang R. Li T. Xiao W. Wang H. (2007). Hydrogen peroxide stimulates macrophages and monocytes to actively release HMGB1. J. Leukoc. Biol. 81, 741–747 10.1189/jlb.0806540 17135572 PMC1808495

[B52] Thomas J. O. (2001). HMG1 and 2: architectural DNA-binding proteins. Biochem. Soc. Trans. 29, 395–401 10.1042/BST0290395 11497996

[B53] Tian J. Avalos A. M. Mao S.-Y. Chen B. Senthil K. Wu H. (2007). Toll-like receptor 9-dependent activation by DNA-containing immune complexes is mediated by HMGB1 and RAGE. Nat. Immunol. 8, 487–496 10.1038/nrg2148 17417641

[B54] Venereau E. Casalgrandi M. Schiraldi M. Antoine D. J. Cattaneo A. De Marchis F. (2012). Mutually exclusive redox forms of HMGB1 promote cell recruitment or proinflammatory cytokine release. J. Exp. Med. 209, 1519–1528 10.1084/jem.20120189 22869893 PMC3428943

[B55] Vettermann C. Castor D. Mekker A. Gerrits B. Karas M. Jäck H.-M. (2011). Proteome profiling suggests a pro-inflammatory role for plasma cells through release of high-mobility group box 1 protein. Proteomics 11, 1228–1237 10.1002/pmic.201000491 21319304

[B56] Wang H. Bloom O. Zhang M. Vishnubhakat J. M. Ombrellino M. Che J. (1999). HMG-1 as a late mediator of endotoxin lethality in mice. Science 285, 248–251 10.1126/science.285.5425.248 10398600

[B57] Wild C. A. Bergmann C. Fritz G. Schuler P. Hoffmann T. K. Lotfi R. (2012). HMGB1 conveys immunosuppressive characteristics on regulatory and conventional T cells. Int. Immunol. 24, 485–494 10.1093/intimm/dxs051 22473704

[B58] Yanai H. Ban T. Taniguchi T. (2012). High-mobility group box family of proteins: ligand and sensor for innate immunity. Trends Immunol. 33, 633–640 10.1016/j.it.2012.10.005 23116548

[B59] Yang D. Chen Q. Yang H. Tracey K. J. Bustin M. Oppenheim J. J. (2007). High mobility group box-1 protein induces the migration and activation of human dendritic cells and acts as an alarmin. J. Leukoc. Biol. 81, 59–66 10.1189/jlb.0306180 16966386

[B60] Zhang L. Yao Y. Dong Y.-Q. Dong N. Yu Y. Sheng Z. (2008). Relationship between high-mobility group box 1 protein release and T-cell suppression in rats after thermal injury. Shock 30, 449–455 10.1097/SHK.0b013e3181672495 18277947

[B61] Zhu X.-M. Yao Y.-M. Liang H.-P. Xu C.-T. Dong N. Yu Y. (2011). High mobility group box-1 protein regulate immunosuppression of regulatory T cells through toll-like receptor 4. Cytokine 54, 296–304 10.1016/j.cyto.2011.02.017 21419643

